# The Relationships between Cerebrospinal Fluid Glial (CXCL12, CX3CL, YKL-40) and Synaptic Biomarkers (Ng, NPTXR) in Early Alzheimer’s Disease

**DOI:** 10.3390/ijms241713166

**Published:** 2023-08-24

**Authors:** Agnieszka Kulczyńska-Przybik, Maciej Dulewicz, Julia Doroszkiewicz, Renata Borawska, Agnieszka Słowik, Henrik Zetterberg, Jörg Hanrieder, Kaj Blennow, Barbara Mroczko

**Affiliations:** 1 Department of Neurodegeneration Diagnostics, Medical University of Bialystok, 15-269 Bialystok, Poland; 2 Department of Psychiatry and Neurochemistry, Institute of Neuroscience and Physiology, The Sahlgrenska Academy at the University of Gothenburg, 405 30 Gothenburg, Sweden; 3 Department of Neurology, Jagiellonian University, 30-688 Kraków, Poland; 4 Clinical Neurochemistry Laboratory, Sahlgrenska University Hospital, 431 80 Mölndal, Sweden; 5 Department of Neurodegenerative Disease, UCL Institute of Neurology, Queen Square, London WC1N 3BG, UK; 6 UK Dementia Research Institute at UCL, London WC1N 3AR, UK; 7 Hong Kong Center for Neurodegenerative Diseases, Clear Water Bay, Hong Kong, China; 8 Wisconsin Alzheimer’s Disease Research Center, University of Wisconsin School of Medicine and Public Health, University of Wisconsin-Madison, Madison, WI 53792-2460, USA; 9 SciLifeLab, University of Gothenburg, 405 30 Gothenburg, Sweden; 10 Department of Biochemical Diagnostics, Medical University of Bialystok, 15-269 Bialystok, Poland

**Keywords:** Alzheimer’s disease, mild cognitive impairment, chemokines, CXCL12, CX3CL1, neurogranin, NPTXR, microglial activation, synaptic damage

## Abstract

In addition to amyloid and tau pathology in the central nervous system (CNS), inflammatory processes and synaptic dysfunction are highly important mechanisms involved in the development and progression of dementia diseases. In the present study, we conducted a comparative analysis of selected pro-inflammatory proteins in the CNS with proteins reflecting synaptic damage and core biomarkers in mild cognitive impairment (MCI) and early Alzheimer’s disease (AD). To our knowledge, no studies have yet compared CXCL12 and CX3CL1 with markers of synaptic disturbance in cerebrospinal fluid (CSF) in the early stages of dementia. The quantitative assessment of selected proteins in the CSF of patients with MCI, AD, and non-demented controls (CTRL) was performed using immunoassays (single- and multiplex techniques). In this study, increased CSF concentration of CX3CL1 in MCI and AD patients correlated positively with neurogranin (r = 0.74; *p* < 0.001, and r = 0.40; *p* = 0.020, respectively), ptau181 (r = 0.49; *p* = 0.040), and YKL-40 (r = 0.47; *p* = 0.050) in MCI subjects. In addition, elevated CSF levels of CXCL12 in the AD group were significantly associated with mini-mental state examination score (r = −0.32; *p* = 0.040). We found significant evidence to support an association between CX3CL1 and neurogranin, already in the early stages of cognitive decline. Furthermore, our findings indicate that CXCL12 might be a useful marker for tract severity of cognitive impairment.

## 1. Introduction

Currently, Alzheimer’s disease (AD) is unquestionably a global disorder, due to its high prevalence worldwide [1]. The long asymptomatic stage of AD does not allow for early diagnosis; on the other hand, it allows for finding early indicators of the disease to track its onset and progression and to seek possible therapeutic targets. The pathogenesis of this neurodegenerative disease is multifactorial and complex [2]. However, well-known, key neuropathological hallmarks of AD are aggregated amyloid beta plaques and phosphorylated tau proteins that build neurofibrillary tangles (NFT), which initiate apoptosis of neurons in the central nervous system (CNS). The neurotoxic deposits of misfolded amyloid β (Aβ) oligomers are known to induce neuroinflammation and neurodegeneration processes that contribute to disease progression [3]. Importantly, it is believed that Aβ aggregates are also involved in neuronal and synaptic dysfunction, which consequently results in problems with learning and memory deficits in AD [4]. The study of the exact mechanisms of microglial activation and synaptic disturbance leading to disease progression and the interrelationships of the proteins involved in these processes have begun to attract more research attention [5].

CXCL12, also referred to as stromal cell-derived factor 1 α (SDF-1α), is one of the major chemokines engaged in the inflammatory process and regulation of the migration of bone marrow-derived microglia cells (BMDM) from blood to the brain. CXCL12 is one of the hematopoietic growth factors (HGFs) and plays a crucial role in the mobilization, adhesion, and differentiation of CD34+ progenitor cells [6,7,8]. The chemokine is also involved in the modulation of synaptic plasticity, through the regulation of neuronal excitability, propagation of signals within glial cells, synaptic transmission, intensification of glutamate release, and inhibition of the accumulation of Aβ through the activation of microglia [4,9,10]. CXCL12 is also known to promote neuroprotective effects and support neurogenesis in the brain via the CXCR4 receptor. Under pathological conditions, CXCL12 protects neurons through the activation of phosphorylation and by triggering nuclear translocation of anti-apoptotic proteins [11,12]. On the other hand, CXCL12 is also a ligand for CXCR7 that appears to participate in the progression of neurodegenerative diseases, including AD [13]. Recent investigations have suggested that overexpression of the *CXCL12 gene* contributes to the pathologic process of AD and could be considered an independent risk factor for AD diagnosis [14]. However, in the brain tissue, cerebrospinal fluid (CSF), and plasma of patients with AD, decreased levels of CXCL12 were found [7,15].

Other pivotal modulators of an inflammatory state in the CNS are CX3CL1 and YKL-40, molecules that regulate microglial and astrocytic activation [16,17,18,19]. Considering the crucial role of the CX3CL1/CX3CR1 axis in microglia–neuron communication, it has been reported that it is involved in microglial activation, neuronal survival, synaptic plasticity, and a variety of synaptic functions, as well as neuronal excitability via cytokine release modulation, chemotaxis, and phagocytosis [20]. Additionally, the CX3CL1 axis regulates plaque load and cognition [21]. Disruption of CX3CL1/CX3CR1 signaling in neurodegenerative diseases, including AD, has been demonstrated [19,22]. The experimental studies provided evidence that disturbance in CX3CL1 signaling may negatively influence the impairment of cognitive function and synaptic plasticity through an increase in action of IL-1β, which may induce NF-κB-dependent transcription of proinflammatory cytokines, such as IL-6 and TNF-α [23]. In AD, CX3CL1 is involved in clearing amyloid plaque and inhibiting intraneuronal accumulation of phosphorylated tau proteins [24].

Neurogranin (Ng) and neuronal pentraxins receptor (NPTXR) are synaptic proteins involved in the regulation of physiological processes of memory and other cognitive functions [25,26]. Increased CSF levels of Ng and decreased CSF levels of NPTXR may be applied as biomarkers of early synaptic dysfunction in AD [27]. Biomarkers that may track this pathological alteration in AD are greatly needed. Current evidence indicates that neurogranin may serve as a marker to predict disease progression [26]. Interestingly, studies concerning NPTXR revealed that CSF concentrations of this protein may be also used as promising biomarkers of AD severity and could inform therapeutic success and disease progression in clinical settings [25].

With these aspects in mind, this study aimed to investigate the relationships between selected indicators reflecting microglial activation, markers of synaptic dysfunction, and amyloid and tau pathology in the early stages of cognitive impairment to identify the role of selected proteins in the progression of the disease. In the literature, there are findings available concerning the influence of proinflammatory factors on synaptic plasticity and the disturbance of neuronal functioning. However, what is crucial seems to be the knowledge of which exact proteins are engaged in this process and interact with each other during the development of this pathomechanism. Therefore, we conducted a comparative analysis of selected proteins related to an inflammatory state in the central nervous system (CNS), such as YKL-40, chemokines—CXCL12 and CX3CL1, with proteins reflecting synaptic damage—Ng, NPTXR, and Aβ 1-42, as well as tau proteins (tau, ptau181) in the MCI and early full-blown AD. To our knowledge, no studies have yet compared CXCL12 and CX3CL1 with markers of synaptic disturbance in the CSF of subjects with early stages of dementia. This study is a continuation of our previous research on the subject of inflammation-induced neurodegeneration and the development of other pathological alterations dependent on the activation of inflammatory states in diseases of the central nervous system [28,29].

## 2. Results

### 2.1. Subject Characterization—Demographics of the Study Participants

A total of seventy-eight newly diagnosed neurological patients were enrolled in this study, including patients with Alzheimer’s disease (*n* = 40), those with mild cognitive impairment (MCI) (*n* = 18), and non-demented controls (*n* = 20) (CTRL). The cohort included 54 women and 24 men, with a distribution of 78%, 56%, and 60% in the AD, MCI, and control groups, respectively. Assessment of cognitive function, performed using the MMSE scale, revealed an expected difference between the AD and MCI groups, as well as Controls (median of MMSE score—22, 27, and 28 points, respectively). There were no significant age differences in the three study groups (*p* = 0.113). The biochemical characteristics of tested markers are summarized in Table 1. All CSF neurochemical dementia biomarkers demonstrated significant differences in concentrations between the three study groups (*p* <  0.001 in the Kruskal–Wallis test).

### 2.2. Quantitate Comparison of the CSF Levels of CXCL12, CX3CL1, YKL-40, Ng, and NPTXR across Diagnostic Groups

We measured the concentrations of selected biomarkers reflecting microglial activation and synaptic dysfunction/damage in early stages of cognitive decline, including early MCI, and early AD dementia. The CSF concentrations of tested markers are presented in Table 2; the significant differences between all tested groups were observed for CXCL12 (*p* < 0.001), CX3CL1 (*p* < 0.001), YKL-40 (*p* = 0.013), NPTXR (*p* = 0.001), and Ng (*p* < 0.001).

**Figure 1:**
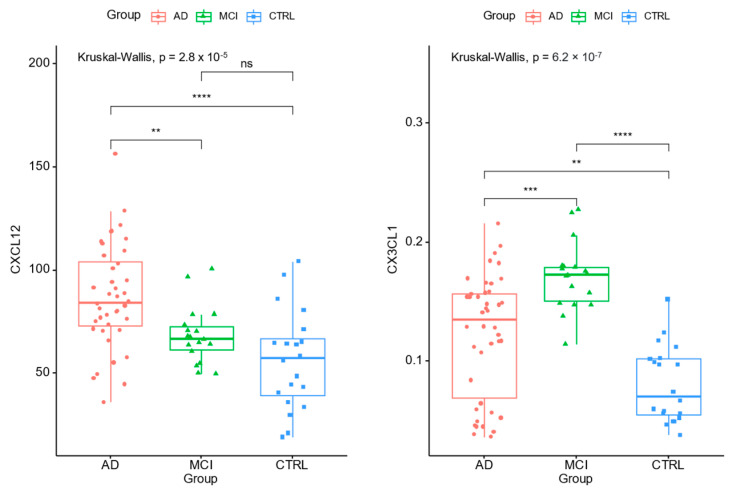
CSF concentrations of CXCL12 and CX3CL1 across diagnostic groups (AD, MCI, CTRL). Statistically significant levels ** *p* < 0.01 *** *p* = 0.001 **** *p* < 0.001, ns—not significant.

Our findings revealed significantly decreased levels of NPTXR as well as increased concentrations of CXCL12, CX3CL1, and YKL-40, and biomarkers of synaptic damage-Ng in MCI and AD patients as compared to controls (Table 2, Figure 1). However, the highest levels of CX3CL1 and YKL-40 were observed in MCI individuals, whereas the concentrations of CXCL12 and Ng increased with the severity of the disease and reached a concentration peak in AD patients. Additionally, significant differences in CXCL12 were observed between AD patients and controls (*p* <  0.001) as well as between AD patients and MCI subjects (*p* =  0.005), but not between MCI subjects and the control group, whereas, for Ng, differences were only observed between AD and controls (*p* <  0.001) (Table 2). Among all tested proteins, statistically significant differences between all study groups were found only for CX3CL1 (MCI vs. CTRL *p* <  0.001; AD vs. CRTL *p* =  0.007; and AD vs. MCI *p* =  0.001) (Table 2). The decreased levels of NPTXR allow for differentiation of dementia patients, as well as those with MCI and AD, from controls (*p* =  0.002 and *p* =  0.013, respectively) (Table 2), but not between both dementia stages (*p* =  0.536).

### 2.3. Correlations between Selected Chemokines, YKL-40, Biomarkers of Synaptic Degeneration, and Core CSF AD Biomarkers

Associations between the tested biomarkers (CXCL12, CX3CL1, YKL-40, Ng, and NPTXR), cognitive status, and neurochemical dementia biomarkers of AD were compared. In the whole study group, the significant association between MMSE and CXCL12 (r = −0.51; *p* < 0.001), Ng (r = −0.35; *p* < 0.001), tau (r = −0.67; *p* < 0.001), pTau181 (r = −0.55; *p* < 0.001), NPTXR (r = 0.24; *p* = 0.030), Aβ42 (r = 0.53; *p* < 0.001), and Aβ42/40 ratio (r = 0.59; *p* < 0.001) was demonstrated. Furthermore, CX3CL1 correlated positively with CXCL12 (r = 0.25; *p* = 0.020), Ng (r = 0.41; *p* < 0.001), Qalb (r = 0.33; *p* < 0.001), tau (r = 0.22; *p* = 0.050), ptau181 (r = 0.29; *p* = 0.010), and YKL-40 (r = 0.34; *p* < 0.001). An increased concentration of CXCL12 was correlated positively with Ng levels (r = 0.36; *p* < 0.001), tau (r = 0.50; *p* < 0.001), and ptau181 (r = 0.43; *p* < 0.001), and negatively with Aβ42 (r = −0.27; *p* = 0.020) and Aβ42/40 ratio (r = −0.45; *p* < 0.001). Additionally, levels of NPTXR were associated with lower concentrations of Aβ42 (r = 0.51; *p* < 0.001). The positive correlation between Ng and tau (r = 0.72; *p* < 0.001), ptau181 (r = 0.76; *p* < 0.001), and YKL-40 (r = 0.48; *p* < 0.001), as well as a negative correlation with Aβ42/40 ratio (r = −0.50; *p* < 0.001), were noticed. An increased concentration of YKL-40 was also associated with classical AD biomarkers: Aβ42/40 ratio (r = −0.24; *p* = 0.030), tau (r = 0.45; *p* < 0.001), and ptau181 (r = 0.58; *p* < 0.001).

**Figure 2:**
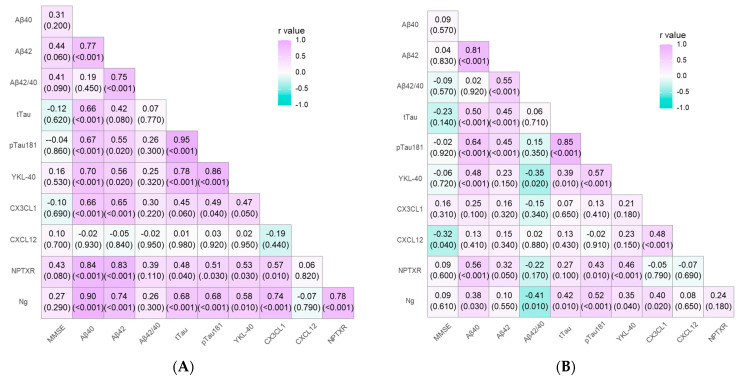
Spearman correlations between CSF proteins, MMSE, as well as amyloid and tau biomarkers in patients with mild cognitive impairment (**A**) and Alzheimer’s disease (**B**). The X and Y axes display biomarkers that correlate with each other. The scale of the colors visualizes the strength of relationships between selected biomarkers: the more intense the color, the stronger the correlation. Additionally, the green color presents negative correlations, whereas purple presents positive correlations.

A significant positive correlation was noticed in the MCI group (Figure 2A) between CX3CL1 and Ng (r = 0.74; *p* < 0.001), ptau181 (r = 0.49; *p* = 0.040), and YKL-40 (r = 0.47; *p* = 0.050). Moreover, Ng levels corelated with tau (r = 0.68; *p* < 0.001), pTau181 (r = 0.68; *p* < 0.001), and YKL-40 (r = 0.58; *p* = 0.010). Decreased levels of NPTXR were associated with lower levels of Aβ42 (r = 0.83; *p* < 0.001). Significant associations between YKL-40 and tau (r = 0.78; *p* < 0.001), as well as ptau181 (r = 0.86; *p* < 0.001), were found.

In the group of patients with AD (Figure 2B), we found that increased concentrations of CXCL12 correlated negatively with MMSE (r = −0.32; *p* = 0.040) and positively with CX3CL1 (r = 0.48; *p* < 0.001). CX3CL1 correlated positively with Ng levels (r = 0.40; *p* = 0.020). In addition, the significant relationships between Ng and tau (r = 0.42; *p* = 0.010), ptau181 (r = 0.52; *p* < 0.001), YKL-40 (r = 0.35; *p* = 0.04), and Aβ42/40 ratio (r = −0.41; *p* = 0.010) were observed. The levels of NPTXR were associated with lower Aβ42 (r = 0.32; *p* = 0.050). Increased concentrations of YKL-40 correlated positively with tau protein (r = 0.39; *p* = 0.010) and ptau181 (r = 0.57; *p* < 0.001), and negatively with Aβ42/40 ratio (r = −0.35; *p* = 0.020).

### 2.4. Diagnostic Performance of Selected Candidate Biomarkers

Diagnostic performance was assessed for biomarkers that display significant differences in the first step of the statistical analysis. The highest clinical accuracy in discrimination between MCI and the control group among tested biomarkers was presented for CX3CL1 (AUC = 0.981, 95% Cl 0.948–1.00, *p* < 0.001). Receiver operating characteristic curve analysis demonstrated that NPTXR and YKL-40 levels also discriminated MCI from controls (AUC = 0.825, 95% Cl 0.681–0.970, *p* < 0.001; and AUC = 0.767, 95% Cl 0.60–0.929, *p* = 0.001, respectively). 

To evaluate the clinical accuracy of potential novel biomarkers in differentiation between AD and controls, the AUC was calculated. The highest AUC values were for Ng and CXCL12 (AUC = 0.890, 95% Cl 0.806–0.974, *p* < 0.001, and AUC = 0.824, 95% Cl 0.710–0.938, *p* < 0.001, respectively). Similar AUC values in discrimination between AD and the control group were demonstrated for CX3CL1 (AUC = 0.738, 95% Cl 0.615–0.861, *p* < 0.001) and NPTXR (AUC = 0.734, 95% Cl 0.610–0.858, *p* < 0.001), and the weakest differentiation performance was shown for YKL-40 (AUC = 0.683, 95% Cl 0.547–0.819, *p* = 0.008).

Among all selected potential biomarkers, only chemokines CX3CL1 and CXCL12 revealed clinical accuracy in discriminating MCI and AD (AUC = 0.788, 95% Cl 0.669–0.907, *p* < 0.001, and AUC = 0.766, 95% Cl 0.637–0.895, *p* < 0.001, respectively).

## 3. Discussion

A growing body of evidence implies that different pathological changes appear before the accumulation of Aβ and tau aggregates. One of the early changes seems to be the oligodendrocyte cell injury with subsequent loss of myelin, as well as the inflammatory state in AD [30]. It is worth emphasizing that chronic and sustained activation of microglia and astrocytes may result in lesions in white matter tracts and destroy the communication between neurons. In AD, chronic secretion of inflammatory cytokines by activated microglia attenuates their ability to dispose of toxic substances from the brain tissue, which may also influence myelin damage. Furthermore, reports from studies on animal AD models indicated that activation of microglia and immunological mechanisms mediate early synaptic dysfunction before amyloid plaque generation. Findings showed that in addition to resident microglia, a different type of microglia is also present in the brain that originates from monocyte precursor cells from bone marrow [4]. The bone marrow-derived microglia cells (BMDM) may pass the blood–brain barrier (BBB) and enter the brain in a chemokine-dependent manner [31]. In AD brains, an accumulation of bone marrow-derived cells was found, and it is suggested that BMDM cells play a pivotal role in internalizing and phagocytizing Aβ [4,31]. One of the chemokines involved in migration cells through the BBB and synaptic disturbance seems to be CXCL12. The investigation of the relationship between the earliest pathogenic processes that may precede the accumulation of amyloid plaques and lead to the development of a full-blown neurodegenerative process seems to be very important in better understanding the biology of this disease. Recent reports suggest that inflammatory processes and synaptic degeneration might occur before the amyloid and tau aggregates. Therefore, studying the interdependencies between these early pathogenetic processes is crucial. To the best of our knowledge, this is the first study comparing CSF levels of CXCL12, CX3CL1, and YKL-40 with markers of synaptic disturbance, such as Ng, NPTXR, and classical neurochemical AD biomarkers in subjects with early stages of dementia.

Our findings showed significantly increased CSF concentrations of both chemokines CX3CL1 and CXCL12 in patients with MCI and AD compared to controls. In agreement with our result are findings from the investigation assessing genes related to immune cells in AD, which revealed upregulated expression of the *CXCL12 gene* in patients with AD compared with the controls [14]. Moreover, in brain tissues of AD patients, it was shown that the levels of expression depend on the localization; significantly higher expression was found in the medial temporal gyrus and superior frontal gyrus in AD compared to non-demented healthy individuals [32]. However, studies with different results than ours are also available [7,15,33]. In two different papers [7,15], a German group of researchers showed a lower concentration of CXCL12 in CSF and plasma patients with AD in comparison to controls. However, there are a few differences between our studies. Firstly, the authors in both of the above-mentioned studies used different kits for quantitative assessment of CXCL12 than we did, and the protocols of our studies were different. Moreover, both kits used (in our study and Laske et al. [7,15]) could include different antibodies, which may also influence the final result. Secondly, the CSF was processed by different procedures including time, temperature, and speed of centrifugation, as well as the freezing temperature before the measurement of the proteins. Additionally, the authors performed experiments on a smaller group of patients in comparison to ours; the study population included 30 AD vs. 30 controls in the first study and 30 AD vs. 15 controls (for CSF analysis in the second study). The second group of researchers demonstrated that CSF concentrations of CXCL12 were significantly higher in patients with amyotrophic lateral sclerosis and multiple sclerosis, but were not altered in AD, spinal muscular atrophy, or frontotemporal dementia [33]. Andres-Benito et al. [33] applied the same kit for the measurement of CXCL12 as the previously mentioned group, Laske et al. [7,15]. However, the protocols of CSF processing were different. Furthermore, we noticed that in the study of the Andres-Benito et al. [33], the population of patients with AD (AD = 19 vs. 44 controls) was about 10 years younger than in our study, which could potentially have an influence on the divergent results of the studies. Since the increased concentrations of this biomarker were also found in other neurodegenerative diseases such as multiple sclerosis (MS), traumatic brain injury (TBI), and amyotrophic lateral sclerosis (ALS), the limitation of this biomarker is thus its lack of specificity [33]. Bearing in mind that CXCL12 acts as a potent chemoattractant for different immunological cells, including monocytes and T and B lymphocytes, we suggest that increased CSF CXCL12 could be related to the activation of the neuroinflammation and local immune response, more specifically, the attraction of leukocytes that migrate along the concentration gradient of chemokine through the blood–brain barrier to their target place. It seems that the source of increased levels of CXCL12 in the CSF might be local production by glial or endothelial cells, or its diffusion and transportation from the blood vessels [34]. The CXCR4-/CXCL12-related underlying mechanism could be that Aβ aggregates activate microglia and an inflammatory cascade leading to secretion of CXCL12, which induces CXCR4-dependent signaling to release pro-inflammatory cytokines both in the microglia and in astrocytes. Inhibition of the CXCR4/CXCL12 axis promotes the translocation of endogenous HSCs from the bone marrow to the blood, facilitating the recruitment of BMDM cells into the brain, and attenuates the neuroinflammation process, which involves the release of excitotoxic markers such as TNFα, intracellular Ca^2+^, and glutamate and increases monocarboxylate transporter 1, the major L-lactate transporter in the brain [10]. Moreover, other researchers postulated that CXCL12 protects neurons from apoptosis and synaptic–dendritic damage caused by AB plaques via activation of PI-3-kinase and ERK pathways and inhibits oxidative damages [35]. Notably, in tissues, CXCL12 is released in pathological conditions, opposite to bone marrow, where this protein and its receptor CXCR4 are constitutively produced to promote the adherence of stem cells to the bone marrow matrix. In damaged brains, increased levels of CXCL12 were revealed. Authors suggested that CXCL12 attracts circulating hematopoietic stem cells (HSCs), which on the surface present expression of CXCR4 and activate inflammation [36]. 

Interestingly, we found a correlation between CXCL12 and Aβ42, as well as the Aβ42/40 ratio in the whole study group, which might indicate the role of this chemokine in clearing Aβ from the CSF. In a previous report, in agreement with our observations, authors suggest that CXCL12 in the CSF may facilitate the migration of antibodies like IgG to the CSF and brain to remove Aβ1-42 from the brain to the plasma by CSF [7]. Moreover, studies of APP/PS1 transgenic mouse brains revealed that administration of CXCL12 after 8 weeks decreased brain Aβ deposition and the concentrations of soluble Aβ-1-40 and Aβ-1-42 by ~50% [4]. The neuroprotective role of CXCL12 was also suggested to be due to a CXCL12-dependent increase in the expression of ADAM-17 that is involved in clearing the plaque burden [37,38]. Based on the significant relationships between CXCL12, Ng, tau, and ptau181 revealed in the whole study group, we assume that this protein may play a role in postsynaptic damage and neurodegeneration. Neuronal damage caused by activated microglia may release tau and Ng in the CSF. Studies carried out on brain tissues of AD patients also confirmed the relationship between CXCL12 and Ng, which implies that both processes reflected by these proteins, i.e., microglial activation and synaptic disturbance, are strictly related [32]. In line with our findings, an analysis of gene expression in AD brains demonstrated significant positive correlations between CXCL12 expression and CSF levels of Aβ-1-42, tau, and ptau181 [14]. Additionally, the correlation between CX3CL1 and ptau181 protein only in MCI subjects may suggest neuroprotective functions of CX3CL1 associated with tau pathology. At disease onset, the levels of CX3CL1 may increase to protect the brain against tau aggregates. This interpretation is also shared by other authors [39,40]. Moreover, our study demonstrated a significant link between NPTXR and Aβ-1-42 in MCI and AD patients; however, at the earliest stages of the disease, the relationship was stronger. In agreement with this, other investigations showed that NPTXR was associated with PET-Aβ imaging and core CSF Aβ42 [41]. These findings may indicate that as a consequence of amyloid pathology, glutamatergic signaling appears impaired.

It is noteworthy that we found a negative correlation between CXCL12 and MMSE score in AD patients, which suggests that CXCL12 could be a valuable marker for the assessment of cognitive impairment severity in patients with AD. Our results are consistent with those of Sanfilippo et al., who found that CXCL12 was associated with cognitive deterioration in an Aβ-related manner [32]. In the available literature, we found only one paper describing the association of CSF level of the protein and the scale of cognitive functions; however, it presents a result opposite to ours [7]. The authors of this study showed no significant correlation between CXCL12 CSF levels and MMSE scores in AD patients. Similarly, a lack of association between plasma levels of CXCL12 and MMSE scores at baseline in AD patients was found, although CXCL12 plasma levels positively correlated with changes in cognitive functions over the time period of 15 months [7]. Although synaptic dysfunction and degeneration are closely linked with clinical disease severity and worsen as patients progress from MCI to AD, we confirmed the correlation of Ng with MMSE score only in the whole study group. These findings may suggest that increased CSF Ng levels could be rather a biomarker of the biological evidence of disease state in the AD continuum than a marker reflecting impairment of cognitive function.

To address whether there is any interplay between selected chemokines, presenting not only inflammatory but also synaptic relationships, we conducted a comparative analysis between markers reflecting such pathology. Increased concentrations of CX3CL1 were significantly associated with Ng levels both in AD and MCI patients. Mounting evidence indicates that CX3CL1/CX3CR1 signaling in microglia is highly important for synaptic plasticity and neuromodulation [20,42,43]. For example, CX3CL1 has been shown to inhibit the maintenance of LTP and reduce spontaneous glutamate release and post-synaptic glutamate currents to facilitate the attenuation of hippocampal synaptic plasticity. Moreover, it suppresses glutamate-mediated calcium influx, particularly in hippocampal neurons. CX3CL1 affects the synaptic functions through the CXCR1 receptor, which is probably expressed on the dendrites of hippocampal neurons [43]. Given the abovementioned facts, the association between CX3CL1 and Ng may arise from the damage and loss of dendrites of hippocampal neurons, which are the source of the receptor for CXCR1 for CX3CL1 and Ng, which is abundantly located in the dendritic spine of hippocampal neurons. Because CXCL12 plays a crucial role in many synaptic functions, including regulation of glutamatergic transmission, modulation of neuronal excitability, and release of neurotransmitters, we suspected a correlation between CXCL12 and Ng, although such a relationship was not revealed. In line with previous studies [17,32], we observed a significant association between CSF levels of YKL-40 and Ng in AD and MCI patients. These results showed that YKL-40 promotes inflammatory response in microglial or astrocytic cells that lead to death and loss of neurons and releasing Ng to the CSF from the dendritic spine. Interestingly, this association seems to be more accelerated in the earliest stages of disease (in MCI) when the levels of YKL-40 in CSF and proinflammatory activities are more augmented.

Although the expression and levels of tested biomarkers have been studied in different neuroinflammatory diseases, there is still no complete information on its possible correlation with other markers of immune response and inflammation. We revealed the relationship between both chemokines CXCL12 and CX3CL1 in AD patients. However, the level of CXCL12 in AD is the highest, whereas the levels of CX3CL1 reach a peak in MCI subjects. Bearing in mind the abovementioned results and the fact that both proteins may have similar functions, including inflammatory and synaptic activity, the conclusion seems to be that these proteins could fulfill different activities based on the other mechanisms depending on the stage of the disease. This hypothesis is also supported by the fact that various inflammatory proteins correlated with each other in AD and MCI subjects; for example, in the MCI group, CX3CL1 was associated with levels of YKL-40, whereas in AD patients, CX3CL1 was associated with CXCL12. Moreover, the relationship of CX3CL1 with Qalb may indicate the role of this protein in immunological response and/or BBB function. We agree with other authors that through augmented releasing of the proinflammatory proteins from the brain to the CSF in the early stages of the disease, neurons attempt to activate microglia to phagocytize pathological aggregates of amyloid β and tau. This postulate is also supported by our findings, which demonstrated the relationship between YKL-40 and Aβ-42 ratio in AD patients. 

Because of some limitations of our study, the findings need to be interpreted carefully. Firstly, in the present study, the number of the investigated population was moderate; however, we chose carefully selected patients with AD, MCI, and controls diagnosed in the same neurological department by experienced neurologists and psychiatrists, regarding clinical data including the history of the patient, neuroimaging examination, neuropsychological tests, biochemical tests, and CSF biomarkers, allowing for biochemical confirmation of the neuropathological process typical for AD. Additionally, samples were stored in the same facilities over similar periods.

Considering the promising results of this investigation, in a future perspective, we would like to continue the study on a larger cohort of patients from different centers. One of our goals will also be to evaluate the effect of treatment on the results of the studied biomarkers; in addition, we would like to compare the concentrations of the studied proteins in the CSF and blood of patients, as well as verify the relationship of the concentrations of the studied proteins in the blood to progressive cognitive impairment. Microglia cells are engaged in synaptic plasticity and normal synaptic functioning in the adult brain; additionally, their removal induces changes in the organization and activity of glutamatergic synapses, the appearance of immature synaptic features, and higher levels of plasticity, which result in the development of cognitive function impairment. However, the exact mechanisms of the actions are still unknown. Thus, it is crucial to understand how microglial activity regulates learning processes and synaptic functions in physiological and pathological conditions.

## 4. Materials and Methods

### 4.1. Study Population

The study population consisted of 78 subjects with neurological disorders (54 women and 24 men, median age of 72 years), who were divided into 3 subgroups: patients with AD (*n* = 40), patients with MCI (*n* = 18), and non-demented subjects, as a control group (*n* = 20) (Table 1). Patients underwent clinical evaluations, neurological, psychiatric, and neuropsychological examinations, neuroimaging tests (magnetic resonance imaging/computed tomography), as well as routine blood and CSF screening assessments. All patients included in the study were in the process of receiving a diagnosis. The diagnosis of MCI and AD was established according to the diagnostic criteria of the National Institute on Aging—Alzheimer’s Association (NIA-AA) [44,45]. To obtain the most accurate clinical diagnosis of AD, neuroimaging and neuropsychological tests were combined with neurochemical AD biomarkers (Aβ1-42, tau, and pTau181 levels and Aβ1-42/Aβ1-40 ratio values). The concentrations of AD biomarkers were interpreted based on the Erlangen Score algorithm [46]. Additionally, we excluded twelve subjects with blood–CSF barrier dysfunction (based on elevated Qalb, which was calculated depending on the age of the patient using the formula for the upper limit of Qalb = [(age/15) + 4] from the study. The median MMSE score in the group of AD patients was 22, with an interquartile range of 19–24 points. The Ethics Committee approved the study at Bialystok University (No. R-I-002/459/2018), and all patients signed an informed consent form before any procedure. The control group consisted of 20 neurological patients (12 females and 8 males) without cognitive impairment. Participants classified for the control group were carefully selected based on neurological, neuropsychological, and laboratory (blood and CSF) tests, which allowed the exclusion of the symptoms’ organic background. Additionally, individuals from the control group did not have subjective memory disorders, did not fulfill the MCI criteria, and had no significant changes in the AD biomarkers (such as Aβ1-42, Aβ1-42/Aβ1-40 ratio, tau, and pTau181). The detailed inclusion and exclusion criteria for non-demented controls were described in our previous paper [47].

### 4.2. Biochemical Measurements

CSF samples were collected into polypropylene tubes through a lumbar puncture. Immediately after collection, the CSF was centrifuged, aliquoted into polypropylene tubes, and stored at −80 °C until processing. All of the tested proteins assessed in the current study were measured using the same batch of reagents. CSF concentrations of biomarkers, such as CX3CL-1, CXCL-12, and YKL-40, were measured using commercially available immunological kits, including the R&D kit (R&D, Minneapolis, MN, USA), Biorbyt kit (Biorbyt, Cambridge, UK), and Quidel kit (Quidel, San Diego, CA, USA), respectively. Biochemical measurements of NPTXR levels were performed with a commercially available RayBioHuman NPTXR ELISA kit (ELH-NPTXR; Ray Biotech, Norcross, GA, USA), whereas Ng concentration was measured with a bead-based immunoassay (MILLIPLEX MAP Human Neuroscience Magnetic Bead Panel 2, HNS2MAG-95K, Merck KGaA, Darmstadt, Germany) using a Luminex^®^ 100/200™ analyzer (Luminex Corporation, Austin, TX, USA). CSF Aβ-42 and Aβ-40 concentrations were analyzed using sandwich ELISA IBL kits (IBL, Hamburg, Germany), while CSF tau and pTau181 levels were quantified using Innotest Fujirebio kits (Europe, Ghent, Belgium). Standards and CSF samples were run in duplicates with a coefficient of variance (CV < 20%). All tests were performed following the manufacturer’s instructions in the Department of Neurodegeneration Diagnostics Medical University of Bialystok. Additionally, the concentration of albumin in the CSF and serum was assessed using the nephelometric method. To evaluate the integrity of the blood–CSF barrier, albumin quotients (Qalb = CSF albumin/serum albumin) were calculated. Individuals with disturbance of the blood–CSF barrier (based on Qalb values) were excluded from the study. 

### 4.3. Data Analysis

The statistical analyses were performed using R 4.2.3 statistical software, with the PMCMRplus package. The three groups were compared with regard to age, gender distribution, MMSE, CSF levels of CXCL-12, CX3CL-1, YKL-40, Ng, and NPTXR, as well as core CSF biomarkers, including Aβ-42, Aβ-40, tau, and pTau181. Nonparametric tests were used since the Shapiro–Wilk test revealed that the concentrations of the tested proteins did not follow a normal distribution. Firstly, a comparison between AD and MCI patients and the control group was performed using the Kruskal–Wallis test. Subsequently, significant differences between the levels of the tested proteins were analyzed using the post hoc Dwass–Steel–Critchlow–Fligner test to verify which groups were different. The results are presented as medians and interquartile ranges. Statistical significance was set at *p* < 0.05. The Spearman correlation coefficient was used for the analysis of relationships between tested variables. The diagnostic performance of potential novel markers was performed using ROC analysis. Power analysis was conducted using unequal sample sizes, an alpha level of 0.01, with Bonferroni correction applied to all group comparisons. These comparisons included assumptions for one-way ANOVA (AD vs. MCI vs. CTRL) as well as for pairs of groups (AD vs. MCI, AD vs. CTRL, MCI vs. CTRL). Importantly, in no instance did the analysis yield a value below 0.8, indicating robust statistical power. Moreover, the power for CX3CL1 and YKL-40 was equal to 1.0, for CXCL12 0.92, for Ng 0.8, and for NPTXR 0.88.

## 5. Conclusions

Understanding the early pathological mechanisms of the development and progression of MCI and AD, particularly synaptic disturbance and neuroinflammatory process, might be crucial for the development of successful, targeted treatment for these diseases. In light of the aforementioned facts, investigations assessing the relationship between different molecules contributing to AD pathogenesis appear to be significant. We found that increased CX3CL1 concentration, a chemokine that affects synaptic plasticity and cognition, correlates with Ng concentration already at an early stage of the disease (MCI). Notably, this association persists across the spectrum of the disease, including those with early AD dementia. Additionally, our findings indicate that CXCL12 might be a useful marker to track the severity of cognitive impairment in patients with AD. Furthermore, CX3CL1 presents the highest clinical accuracy in discriminating MCI from controls and AD patients, although Ng and CXCL12 seem to be better at differentiating between AD and controls.

## Figures and Tables

**Table 1:** Biochemical characteristics of the investigated groups—CSF biomarker levels.

Variables in CSF	Group	Aβ-42/40	Tau [pg/mL]	pTau181 [pg/mL]
Median	MCI	0.045	389	57
	AD	0.030	669	83
	CTRL	0.066	222	37
25th percentile	MCI	0.037	327	47
	AD	0.028	572	69
	CTRL	0.057	189	33
75th percentile	MCI	0.058	495	68
	AD	0.037	897	109
	CTRL	0.076	272	42
*p*-value Dwass–Steel test	AD vs. CTRL	<0.001	<0.001	<0.001
	AD vs. MCI	<0.001	<0.001	<0.001
	MCI vs. CTRL	0.007	<0.001	<0.001

**Table 2:** Concentrations of tested proteins in cerebrospinal fluid patients with AD and MCI and subjects from the control group.

Tested Variable	Median (Interquartile Range)			*p* (KW-Test)	*p* (Dwass–Steel–Critchlow–Fligner Test)		
	AD	MCI	Controls		AD vs. CTRL	AD vs. MCI	MCI vs. CTRL
CXCL12 [pg/mL]	84 (73–104)	67 (61–73)	57 (39–67)	<0.001	<0.001	0.005	0.147
CX3CL1 [pg/mL]	135 (70–160)	172 (150–180)	71 (50–100)	<0.001	0.007	0.001	<0.001
YKL-40 [ng/mL]	399 (272–520)	418 (315–513)	292 (233–369)	0.013	0.049	0.748	0.012
NPTXR [pg/mL]	15 (11–18)	14 (10–15)	19 (17–22)	0.001	0.013	0.536	0.002
Ng [ng/mL]	822 (663–1166)	695 (490–841)	487 (435–580)	<0.001	<0.001	0.072	0.109

## Data Availability

The data presented in this investigation are available on request from the corresponding author. Key data are stated in the text.
